# Sishen Pill Ameliorates Dextran Sulfate Sodium (DSS)-Induced Colitis with Spleen-Kidney Yang Deficiency Syndromes: Role of Gut Microbiota, Fecal Metabolites, Inflammatory Dendritic Cells, and TLR4/NF-*κ*B Pathway

**DOI:** 10.1155/2022/6132289

**Published:** 2022-10-03

**Authors:** Wei Ge, Bu-Gao Zhou, You-Bao Zhong, Su-Qing Liu, Jia-Qi Huang, Wang-Yuan Yuan, Chang-Ying Xie, Duan-Yong Liu, Hai-Yan Wang, Zheng-Yun Zuo

**Affiliations:** ^1^Department of Proctology, Affiliated Hospital of Jiangxi University of Chinese Medicine, Nanchang 330006, China; ^2^Department of Postgraduate, Jiangxi University of Chinese Medicine, Nanchang 330004, China; ^3^Formula-Pattern Research Center, Jiangxi University of Chinese Medicine, Nanchang 330004, China

## Abstract

Sishen pill (SSP) is an old Chinese medicine used to treat colitis with spleen-kidney-yang deficiency (SKYD) syndromes. However, its exact mechanism of action has not yet been fully elucidated. The aim of this study was to evaluate the effects and potential mechanisms of SSP on colitis with SKYD syndromes in mice. Colitis with SKYD syndromes was induced by rhubarb, hydrocortisone, and dextran sulfate sodium (DSS), and treatment was provided with SSP. Flow cytometry was performed to examine the inflammatory dendritic cell (infDC) regulations of SSP. The changes in the gut microbiota (GM) and fecal metabolites post-SSP treatment were investigated using the combination of 16S rRNA sequencing and untargeted metabolomics. Additionally, we also examined whether SSPs could regulate the infDCs by modifying TLR4/NF-*κ*B signaling pathways. Compared with the DSS group, the disease activity index, colonic weight, index of colonic weight, and colonic injury scores, as well as the levels of tumor necrosis factor (TNF)-*α*, interleukin (IL)-1*β*, IL-6, and IL-12p70 decreased significantly in the DSS + SSP group, while free triiodothyronine (FT3), free tetraiodothyronine (FT4), testosterone (TESTO), body weight change, colonic length, and the levels of IL-10 increased. Also, SSP decreased the amounts of CD103^+^CD11c^+^iNOS^+^, CD103^+^CD11c^+^TNF-*α*^+^, CD11c^+^CD103^+^CD324^+^, CD103^+^CD11c^+^MHC-II^+^, and CD103^+^CD11c^+^CD115^+^. Interestingly, 16S rRNA sequencing and untargeted metabolomics showed that SSP treatment restored the dysbiosis of GM and improved the dysfunction in fecal metabolism in colitis mice with SKYD syndromes. Correlation analysis indicated that the modulatory effects of SSP on FT3, FT4, IL-10, colonic weight index, CD103^+^CD11c^+^TNF-*α*^+^, CD103^+^CD11c^+^MHC-II^+^, and 13 common differential metabolites were related to alterations in the abundance of *Parvibacter, Aerococcus, norank_f_Lachnospiraceae, Lachnospiraceae_UCG-006, Akkermansia,* and *Rhodococcus* in the GM. In addition, SSP markedly inhibited the activation of the TLR4, MyD88, TRAF6, TAB2, and NF-*κ*Bp65 proteins and activated I*κ*B. These results indicate that SSP can effectively alleviate colitis mice with SKYD syndrome by regulating infDCs, GM, fecal metabolites, and TLR4/NF-*κ*B signaling pathways.

## 1. Introduction

Inflammatory bowel disease (IBD), including ulcerative colitis (UC) and Crohn's disease, is a chronic and nonspecific inflammatory disease of unknown etiology affecting the gastrointestinal tract [1]. With accelerated global industrialization, the incidence of IBD has stabilized in the West, while its incidence has been gradually increasing in newly industrialized countries [2]. Unfortunately, there is currently no cure for IBD.

As early as the 1860s, the importance of microorganisms for developing jejunal and ileal crypt structures in rodents was first reported [3]. A growing number of studies have confirmed that the dysbiosis of the gut microbiota (GM) may be a cause or consequence of the pathogenesis of IBD [4]. Certain bacteria in the phyla *Actinobacteria*, *Bacteroidetes*, *thick-walled bacteria*, and *Aspergillus* have been strongly associated with IBD [5]. In addition, some studies suggest that the modulation of GM using probiotics has beneficial effects on IBD [6].

As an intermediate link between GM and host metabolism, the metabolites of GM have a key role in promoting intestinal homeostasis. The dysregulation of lipid metabolism is a typical feature of IBD pathogenesis. Previous studies have found that CerPI, CerPE, and dihydroceramide production by *Bacteroides* in feces are significantly lower in the UC population than in the non-IBD population [7]. Another study suggested that total short-chain fatty acids and butyrate concentrations are negatively correlated with the intestinal inflammatory response induced by *Salmonella* [8]. These studies suggest microbial metabolites provide more targets for the treatment of IBD, and the combination of 16S rRNA sequencing and metabolomics could provide a reliable method for elaborating the mechanisms of Chinese herbal formulas via the interactions between GM and host metabolism [9].

The function of dendritic cells (DCs) in innate immunity should not be ignored in IBD. DCs ensure intestinal homeostasis by maintaining immune tolerance to nutritional and commensal microbiota in a healthy gut, while an abnormal DCs response can lead to intestinal inflammation [10]. The pattern recognition receptor responses of DCs are critical for regulating interactions between innate and adaptive immunity, cytokines, and microbiota in IBD [11]. Some studies have also reported that various probiotics and their metabolites can suppress the inflammatory response by regulating DCs maturation and producing tolerant DCs, which is also considered a new approach for treating IBD [12].

Traditional Chinese medicine (TCM), including herbs, herbal formulas, and acupuncture, has shown to be effective as an alternative and complementary treatment for IBD [13, 14]. Sishen pill (SSP) is a Chinese drug used to treat colitis with spleen-kidney-yang deficiency (SKYD) syndrome. Its major advantages are its clinical efficacy, stability, and low price. Patients with colitis treated with SSP are clinically characterized by persistent or recurrent episodes of dawn diarrhea because of kidney-yang deficiency and spleen-yang deficiency [15]. A clinical study on 87 patients of UC with SKYD syndromes found that the clinical cure rate of SSP was 69.77%, while the clinical cure with sulfasalazine was 40.91% [16]. Our previous studies have confirmed that SSP could effectively alleviate DSS-induced acute colitis [17]. However, according to TCM, establishing the therapeutic relationship between SSP and colitis with SKYD syndromes would better reflect correspondence between the prescription and the syndrome, which is a precise strategy for TCM treatment and a theoretical basis for studying the mechanism of the Chinese medicine formulas. Therefore, in the present study, a mouse model of colitis with SKYD syndromes was prepared to study the effects and potential mechanisms of SSP on colitis with SKYD syndromes.

## 2. Materials and Methods

### 2.1. Mice

Male BALB/c mice, aged 9–12 weeks, weighing 20–22 g, were provided by the Hunan Silaike Jingda Experimental Animal Co. Ltd. (Changsha, China) (Certificate number: SCXK 2017–0004). All animals were housed in an SPF environment with a temperature of 22 ± 1°C, a relative humidity of 50 ± 1%, and a light/dark cycle of 12/12 hr. All animal studies (including the mice euthanasia procedure) were performed in compliance with the regulations and guidelines of the Jiangxi University of Chinese Medicine institutional animal care and conducted according to the AAALAC and the IACUC guidelines.

All animals were acclimatized for 3 days before the experimental studies were performed. Thirty-two mice were then divided into three groups (eight mice/group): normal mice without treatment (Nor group), normal mice treated with SSP (Nor + SSP group), colitis model without treatment (DSS group), and colitis model treated with SSP (DSS + SSP group). Colitis was induced by rhubarb + hydrocortisone + dextran sulfate sodium (DSS).

### 2.2. Drugs

SSP (batch number 17080051) was purchased from Tongrentang Natural Medicine Co. Ltd. (Beijing, China) and was composed of *Psoralea corylifolia* L., *Myristica fragrans* Houtt., *Schisandra chinensis* (Turcz.) Baill, *Euodia rutaecarpa* (Juss.) Benth., *Zingiber officinale* Rosc., and *Ziziphus jujuba* Mill, which were prepared into pills according to the dose ratio (400, 200, 200, 100, 200, and 200 g, ratio: 4 : 2:2 : 1:2 : 2, respectively). The quantitative determination of SSP was analyzed by HPLC-ESI-MS/MS [18], which confirmed that SSP contains deoxyschizandrin (72.6 *μ*g/g), *γ*-schizandrin (131.5 *μ*g/g), schizandrin (258.0 *μ*g/g), schizandrol B (71.2 *μ*g/g), schisantherin A (25.1 *μ*g/g), psoralen (1310.8 *μ*g/g), isopsoralen (1293.7 *μ*g/g), evodiamine (22.2 *μ*g/g), and rutaecarpine (24.0 *μ*g/g). In this study, the manufacturer and batch number of SSP were the same as that used in our previous study. DSS (molecular weight: 36–50 kDa) was obtained from MP Biomedicals (Santa Ana, CA, USA). Other drugs/reagents were from rhubarb (batch Number 8066073), Yifang Pharmaceutical Co. Ltd. (Guangdong, China); hydrocortisone injection (national medicine permission number H41020789), and Runhong Pharmaceutical Co. Ltd. (Henan, China).

### 2.3. Rhubarb + Hydrocortisone + DSS-Induced Experimental Colitis

Animal experiments have confirmed that high oral doses of rhubarb can increase stool quantity and intestinal advance rate, and increase the water content of feces in rodents, inducing symptoms similar to spleen-yang deficiency, such as increased frequency of defecation and loose stools [19]. Moreover, large intramuscular doses of hydrocortisone can decrease body weight and body temperature, as well as the serum level of free triiodothyronine (FT3), free tetraiodothyronine (FT4), and testosterone (TESTO) in rodents, inducing symptoms similar to kidney-yang deficiency, such as emaciation, dispiritedness, aversion to cold, and anorexia [20]. In this study, the animal model of SKYD syndromes was successfully replicated following a previous protocol [21].

Experimental colitis was induced by administrating 1 ml rhubarb solution (12.5 g/kg raw rhubarb granules dissolved in water drunk) by gavage (days 1–7). Subsequently, 25 mg/kg hydrocortisone injection was injected into the buttocks of mice (days 8–14). Mice were then given 3% (wt/vol) dexglycan sodium sulfate (DSS) dissolved in deionized water (days 8–14). Fresh DSS solutions and rhubarb solutions were prepared every morning.

The mice in the Nor group received normal drinking water. Body weight, stool body weight, consistency, intestinal bleeding, hunchback posture, hair standing, and spirit were monitored daily (at 9am).

### 2.4. Therapeutic Protocols

**Figure 1:**
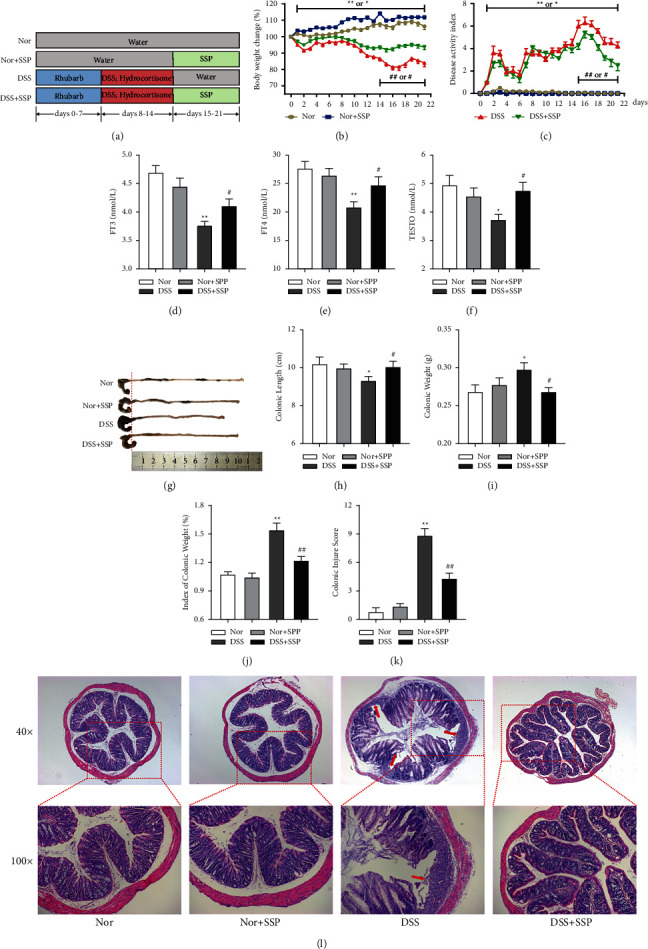
Therapeutic effect of SSP on rhubarb + hydrocortisone + DSS-induced colitis with spleen-kidney yang deficiency syndrome in mice. (a) Experimental protocol. (b) The ratio of day weight to initial body weight. (c) The disease activity index measured every day. (d) Free triiodothyronine (FT3). (e) Free thyroxine (FT4). (f) Testosterone (TESTO). (g) Changes in colonic length by the naked eye. (h) Colonic length. (i) Colonic weight. (j) Index of colonic weight. (k) Colonic injury. (l) Hematoxylin and eosin staining of the colon. Data were presented as means ± SEM (*n* = 8). ^*∗*^*p* *<* *0*.05 and ^*∗∗*^*p* < 0.01 versus Nor group; ^#^*p* < 0.05 and ^##^*p* < 0.01 versus DSS group.

On day 15, according to our previous studies (Figure 1(a)) [17], the Nor + SSP group and the DSS + SSP group were treated orally with SSP (2.5 g/kg/day) dissolved in physiological saline for 7 consecutive days; the DSS and Nor groups were treated with an equal volume of saline. On day 22, all mice were sacrificed under sodium pentobarbital (20 mg/kg i.p.) anesthesia, and the peripheral blood and colon were rapidly separated.

### 2.5. Clinical and Macroscopic Evaluation

The body weight change and disease activity index (DAI) were established according to body weight, stool consistency, and intestinal bleeding daily. The body weight change = current weight/original weight × 100%. The DAI score was established according to standard parameters including body weight loss (0: <1%; 1: 1–5%; 2: 5–10%; 3 : 10–20%; and 4: >20%), stool consistency (0, normal; 2, loose stools; 4, diarrhea), and intestinal bleeding (0, negative; 2, positive; 4, bleeding). The colon length was measured, and the index of colon weight was calculated as follows: the index of colon weight = colon weight/body weight × 100%. The histological injury score was established according to the criteria reported by Schmidt et al. [22]. The total score included inflammatory cell infiltration and tissue injury.

### 2.6. Histological Analysis

The separated colon tissue was fixated in 4% paraformaldehyde solution for 24 h, embedded in paraffin, and cut into 4-*μ*m-thick sections. Paraffin-embedded sections were deparaffinized and rehydrated, followed by staining with hematoxylin and eosin, and evaluated under an Olympus microscope. The histological grading of colitis was as described by Dieleman et al. [23].

### 2.7. Immunochemiluminometric Assay (ICMA)

The blood samples (*n* = 8) were centrifuged for 10 min at 13,000 rpm and 4°C to obtain serum. The levels of FT3 (58594705), FT4 (59321905), and TESTO (62073401) were then detected using commercial ICMA kits (Roche Diagnostics, LA, USA) according to the manufacturer's instructions.

### 2.8. Enzyme-Linked Immunosorbent Assay (ELISA)

The colon tissue (*n* = 8) in the −80°C cryopreservation tube was removed. The RIPA buffer was then added to lyse the tissue at a ratio of 1 : 10, followed by homogenization with an ultrasonic homogenizer on ice, incubation for 30 min at 4°C, and centrifugation for 10 min at 13,000 rpm and 4°C to obtain tissue supernatant. The levels of tumor necrosis factor (TNF)-*α* (88-7324-88), interleukin (IL)-1*β* (88-7013-88), IL-6 (88-7064-88), IL-10 (88-7105-88), and IL-12p70 (88-7121-88) were measured by commercial ELISA kits (Thermo Fisher Scientific, Waltham, MA, USA). Then, the absorbance at 450 nm was determined using a microplate reader (Bio-Rad, Hemel Hempstead, United Kingdom).

### 2.9. Flow Cytometry

The fresh spleen from each mouse was cut, ground, filtered, and centrifuged, and the supernatant was removed to collect red blood cells. Red blood cells were lysed with 1 mL lysis buffer (BD Biosciences, Franklin Lakes, NJ, USA) to obtain splenic blood mononuclear cells. The cell samples were cultured in RPMI-1640 (10% fetal calf serum (FBS), 100 mg/mL gentamycin, 100 U/mL penicillin, and 2 mM L-glutamine), followed by stimulation with a leukocyte activation cocktail (InvivoGen, 00-4975-93) at 37°C in 5% CO_2_ for 2 h. Then, the cells were fixed and permeabilized with a Cytofix/Cytoperm Kit (BD Biosciences, Franklin Lakes, NJ, USA) before the standard surface and intracellular staining procedures. Subsequently, the cells were incubated with antibodies for 40 min at 4°C in the dark. Finally, cells were detected using FACSCanto II flow cytometry (BD Biosciences, Franklin Lakes, NJ, USA). The following mAbs were used: APC rat anti-mouse CD103 (No. 562772) (1 : 100), PE anti-mouse CD107b (553324) (1 : 100), APC-Cy7 rat anti-mouse TNF-*α* (No. 506324), and PerCP-CY5.5 rat anti-mouse CD11c (No. 560584) (1 : 100) (BD Biosciences, Franklin Lakes, NJ, USA); PE rat anti-mouse iNOS (12-5920-80) (1 : 100) and PE anti-mouse CD324 (53-3249-82) (1 : 100) (Invitrogen, Carlsbad, California, USA); and APC-Cy7 rat anti-mouse MHC-II (107628) (1 : 100) and PE anti-mouse CD115 (135524) (1 : 100) (1 : 100) (BioLegend, San Diego, California, USA). Limits for the quadrant markers were set based on negative populations and isotype controls. The data were analyzed by FlowJo software 10 (Tree Star, Ashland, OR, USA), and the inactive cells were excluded by gating.

### 2.10. Fecal DNA Extraction, PCR Amplification, and Illumina MiSeq Sequencing

Colonic contents from all mice were collected and preserved at −80°C. DNA extraction, PCR amplification, and Illumina MiSeq sequencing were conducted using the Majorbio Bio-Pharm Technology (Shanghai, China). Microbial community genomic DNA was extracted from fecal samples using the QIAamp DNA Stool Mini Kit (Qiagen, Valencia, CA, USA). After completing the detection of DNA extraction quality and DNA concentration and purity, the extracted DNA was amplified by an ABI GeneAmp® 9700 PCR thermocycler (ABI, CA, USA) through the hypervariable region V3-V4 of the bacterial 16S rRNA gene. The following primers were used: 338F (5′-ACTCCTACGGGAGgCAGCAGcagg-3') and 806R (5′-GGACTachVGGGTWTCTAat-3′). PCR was performed using TransGen AP221-02: *TransStart* FastPfu DNA Polymerase 20 *μ*L mixtures: 4 *μ*L 5 × *TransStart* FastPfu buffer, 2 *μ*L 2.5 mM dNTPs, 0.8 *μ*L forward primer (5 *μ*mol/L), 0.8 *μ*L reverse primer (5 *μ*mol/L), 0.4 *μ*L FastPfu DNA Polymerase, 10 ng template DNA, and finally ddH_2_ O up to 20 *μ*L. After the PCR was repeated three times, the PCR product was extracted from 2% agarose gel and purified. Finally, the PCR product was detected by 2% agarose gel electrophoresis and quantified using Quantus™ Fluorometer (Promega, USA).

Sequencing was carried out on an Illumina MiSeq PE300 platform/NovaSeq PE250 platform (Illumina, San Diego, USA) according to the standard protocols by Majorbio Bio-Pharm Technology Co. Ltd. (Shanghai, China). The raw reads were deposited into the NCBI Sequence Read Archive (SRA) database (Accession number: PRJNA655607). Operational taxonomic units (OTUs) with 97% similarity cutoff were clustered using UPARSE (version 7.0.1090), and chimeric sequences were identified and removed. The taxonomy of each OTU representative sequence was analyzed by the RDP classifier (http://rdp.cme.msu.edu/, version 2.2) against the SILVA 16S rRNA database (version 123) using a confidence threshold of 0.7. Venn diagrams made using R programming language (version 3.3.1) reflect similarities and overlaps in species OTU levels. A community histogram drawn to determine the species composition of different groups at the level of genus classification (http://www.ggtern.com/) was used to intuitively show the distribution proportion and relationship of genus-level species in the three groups of samples. Linear discriminant analysis effect size (LEfSe) (http://huttenhower.sph.harvard.edu/galaxy/root?tool_id=lefse_upload) measurements (based on the nonparametric factorial Kruskal–Wallis sum-rank test and the Wilcoxon rank-sum test) were used to identify species that were significantly different (from phylum to genus level) among two groups or more groups, with *p* < 0.05 and an linear discriminant analysis (LDA) score threshold of 2. Correlation heatmap analysis calculated the correlation coefficients (Spearman rank correlation coefficient and Pearson correlation coefficient) between environmental factors and species with differences species by R programming language (version 3.3.1) and visually displayed the obtained numerical matrix through the heatmap.

### 2.11. Fecal Sample Preparation and UHPLC-MS/MS Analysis for Metabolomics

A 50-mg fecal sample was accurately weighed, and a 400 *μ*L methanol: water (4 : 1, v/v) solution was added to extract fecal metabolites. The mixture was treated by a high-throughput tissue crusher Wonbio-96c (Shanghai Wanbo biotechnology co., LTD) (50 kHz, −20°C, 6 min), followed by vortexing (30 s), ultrasound (40 kHz, 5°C, 30 min), and precipitation at −20°C for 30 min. After the mixtures were centrifuged at 13,000 rpm for 15 min at 4°C to precipitate the protein, the supernatant was transferred to sampling vials for LC-MS/MS analysis. An in-house quality control (QC) was prepared by pooling and mixing the same volume of each sample. The QC samples were disposed and tested in the same manner as the analytic samples. It helped to represent the whole sample set, which would be injected at regular intervals (every 6 samples) in order to monitor the stability of the analysis. UHPLC analysis was performed on the ExionLC™AD system (AB Sciex, USA) equipped with an ACQUITY UPLC system and with an ACQUITY UPLC BEH C18 column (100 mm × 2.1 mm i.d., 1.7 *μ*m; Waters, Milford, USA). The mobile phase consisted of A, 0.1% formic acid in the water and B, 0.1% formic acid in acetonitrile: isopropanol (1 : 1, v/v). The solvent gradient changed according to the following conditions: 95%–80% A at 0–3 min, 80%–5% A at 3–9 min, 5%–5% A at 9–13 min, 5%–95% A at 13–13.1 min, and 95%–95% A at 13.1–16 min for equilibrating the systems. The column temperature was maintained at 40°C. The flow rate was set to 0.4 mL/min, and the sample injection volume was 20 uL. During the period of analysis, all these samples were stored at 4°C. A quadrupole-time-of-flight mass spectrometer (Triple TOF™5600+, AB, Sciex, USA) was adapted to detection peaks, and the detection was operated in positive mode and negative mode. The optimal conditions were set as follows: both Ion Source GSs were set as follows—GS1 and GS2, 50 psi; curtain gas, 30 psi; source temperature, 500°C; ion-spray voltage floating, −4000 V in negative mode and 5000 V in positive mode; collision energy, 20–60 V rolling; and declustering potential, 80 V for MS/MS. The full MS scan mode was monitored at a mass range of 50–1000 m/z. After UHPLC-MS/MS analyses, the raw data missing value filtering, missing value recoding, data normalization, QC validation, and data transformation. A multivariate statistical analysis was performed using ropls (version 1.6.2) *R* packages. Principle component analysis (PCA) and orthogonal partial least squares discriminate analysis (OPLS-DA) after mean-centering and pareto scaling were performed on the normalized data, and the variable importance in the projection (VIP) value > 1.5, S-plot, and Student's *t*-test*p*-value < 0.05 were used to screen common differential metabolites among two groups. Clustering heat maps and VIP bars showed the significance and expression trends of common differential metabolites. Based on a database search (http://www.genome.jp/kegg/), these metabolites were classified according to the pathways involved or their function. Enrichment analysis was usually used to analyze a group of metabolites in a function node, whether they appeared or not. Scipy.stats (*Python* packages) (version 1.0.0) was exploited to identify statistically significantly enriched pathways using Fisher's exact test. Correlation analysis was performed to calculate the Pearson correlation coefficient between microbiology and differential metabolites.

### 2.12. Western Blot Analysis

TLR4 is overexpressed in colonic mucosal DCs of patients with IBD [13]. So the protein samples of colonic mucosa were prepared as described for ELISA. The protein concentration was determined by a BCA protein assay kit (CoWin Biotech, Jiangsu, China). Equal weight of protein per sample was separated using 10% SDS-PAGE (CoWin Biotech, Jiangsu, China) and transferred to PVDF membranes (Millipore, Billerica, MA, USA). These membranes were blocked at room temperature for 1 h and then incubated overnight with the following primary antibody at 4°C, including anti-GAPDH (No. ab181602) (1 : 2000), TLR4 (No. ab13556) (1 : 500), MyD88 (No. ab2064) (1 : 1000), TRAF6 (No. ab40675) (1 : 5000), TAB2 (No. 3745S) (1 : 1000), I*κ*B (No. 4814S) (1 : 1000), and NF-*κ*Bp65 (No. ab16502) (1 : 1000). All antibodies were purchased from Abcam (Cambridge, UK) except I*κ*B and TAB2 from Cell Signaling Technology (Boston, USA). Next, samples were incubated with secondary antibodies for 1 h at room temperature. The immunoreactive bands were assessed using the ECL substrate (Solarbio, Beijing, China). The images were captured using the Highly Sensitive Chemiluminescence Imaging system (UVP ChemStudio 515, Analytik Jena, Germany). The images were analyzed using Image-Pro Plus 6.0 (Media Cybernetic, USA).

### 2.13. Statistical Analysis

Data were presented as mean ± SEM. The statistical analysis and data visualization were performed using R programming language 3.3.1 and GraphPad Prism 8.0 software (La Jolla, CA, USA). One-way analysis of variance followed by the Tukey-Kramer post hoc test, Student's unpaired *t*-test, and nonparametric factorial Kruskal–Wallis sum-rank test were used to evaluate statistical differences among groups. A *p*-value <0.05 was considered statistically significant. Correlation analyses were conducted by Spearman rank correlation and Pearson correlation test with correlation coefficient >0.5 and *p* < 0.05 marked as significantly relevant.

## 3. Results

### 3.1. SSP Ameliorates UC with SKYD Syndrome in Mice

DSS-induced colitis is one of the classical IBD models in which acute colitis is induced in C57BL/6 mice by ad libitum consumption of 2–3% (w/v) DSS solution for 7 days [24]. In TCM, weakness symptoms such as emaciation, aversion to cold, dispiritedness, and diarrhea can be induced in rodents by using bitter-cold Chinese herbal medicine combined with western drugs that inhibit the hypothalamic-pituitary-adrenal axis [25], which is similar to the clinical manifestations of colitis with SKYD syndromes. Therefore, in this study, we used rhubarb (gavage) + hydrocortisone (intramuscular injection) + DSS (drinking) to induce colitis with SKYD syndromes in mice. Compared with the Nor group, mice in the DSS group showed SKYD syndromes, such as emaciation, dispiritedness, hair erect and back arched, huddle, loose stools, decreased body weight change (Figure 1(b)) and levels of FT3 (Figure 1(d)), FT4 (Figure 1(e)), and TESTO (Figure 1(f)), and increased DAI (Figure 1(c)). Besides, mice in the DSS group showed severe intestinal damage, including shortened colonic length (Figures 1(g) and 1(h)), increased colonic weight (Figure 1(i)), and enhanced index of colonic weight (Figure 1(j)) and colonic injury scores (Figure 1(k)). Histopathological images (Figure 1(l)) further showed the number of inflammatory cells infiltrated into the submucosa of the colon in the DSS group, accompanied by massive destruction of the crypt and ulcer formation. This evidence indicated that the mice showed the pathological manifestations of colitis and the clinical manifestation of SKYD syndromes.

Compared with the DSS group, DAI (Figure 1(c)), colonic weight (Figure 1(i)), index of colonic weight (Figure 1(j)), and colonic injury scores (Figure 1(k)) decreased significantly in the DSS + SSP group, while FT3 (Figure 1(d)), FT4 (Figure 1(e)), TESTO (Figure 1(f)), body weight change (Figure 1(b)), and colonic length (Figures 1(g) and 1(h)) increased significantly. Histopathological images (Figure 1(l)) further showed that colonic mucosa was intact, the crypt structure was partially restored, and there was a small amount of inflammatory cell infiltration in the DSS + SSP group. However, there were no significant differences in the above indexes between the Nor group and the Nor + SSP group. The above results indicate that SSP alleviates rhubarb + hydrocortisone + DSS-induced colitis with SKYD syndromes.

### 3.2. SSP Regulates the Expression of Inflammatory Cytokine in Colitis Mice with SKYD Syndromes

**Figure 2:**
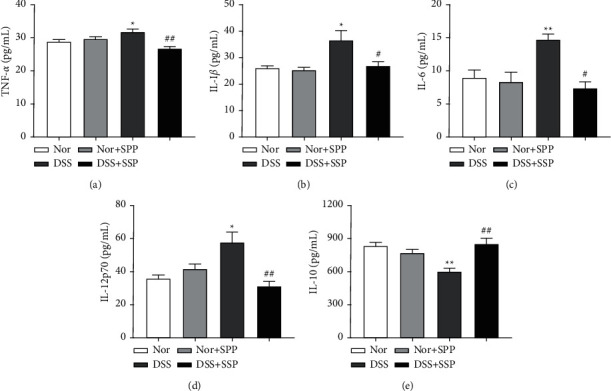
SSP regulates the expression of cytokine in the colonic tissue of colitis mice. (a) TNF-*α* expression; (b) IL-1*β* expression; (c) IL-6 expression.; (d) IL-12p70 expression; (e) IL-10 expression. Data were presented as means ± SEM (*n* = 8). ^*∗*^*p* < 0.05 and ^*∗∗*^*p* < 0.01 versus Nor group; ^#^*p* < 0.05 and ^##^*p* < 0.01 versus DSS group.

Intestinal tissue damage in IBD is mediated by cytokines produced in the inflammatory microenvironment, and blocking cytokine function, such as TNF-*α*, IL-1*β*, IL-6, and IL-12, is a major target of therapeutic intervention in IBD [26]. The levels of TNF-*α* (Figure 2(a)), IL-1*β* (Figure 2(b)), IL-6 (Figure 2(c)), and IL-12p70 (Figure 2(d)) in colon tissue were significantly higher in the DSS group than those of the Nor group, while the levels of IL-10 (Figure 2(e)) were significantly lower. After 7 days of SSP treatment, the levels of TNF-*α* (Figure 2(a)), IL-1*β* (Figure 2(b)), IL-6 (Figure 2(c)), and IL-12p70 (Figure 2(d)) decreased, and the levels of IL-10 (Figure 2(e)) increased. The above data suggest that SSP may inhibit inflammation by regulating the balance between pro-inflammatory cytokines and anti-inflammatory factors in colonic tissues.

### 3.3. SSP Inhibits the Differentiation of Inflammatory DCs in Colitis Mice with SKYD Syndromes

**Figure 3:**
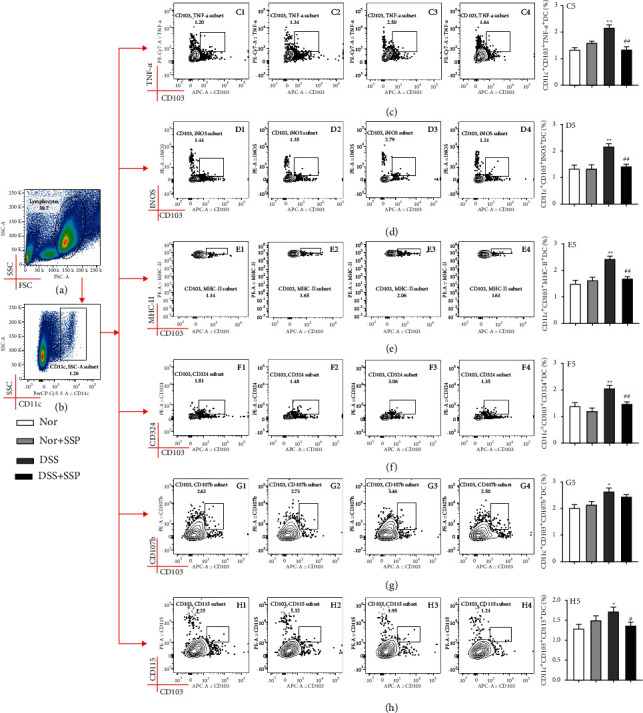
SSP inhibits the differentiation of inflammatory DCs in colitis mice. (a) Ratio of lymphocytes. (b) Percentage of CD11c^+^DCs in lymphocytes. (c) CD11c^+^ CD103^+^TNF-*α*^+^DCs (TNF-*α*^+^DCs) analysis: C1: Nor group; C2: Nor + SSP group; C3: DSS group; C4: DSS + SSP group; C5: statistical analysis of CD11c^+^ CD103^+^TNF-*α*^+^DCs proportion. (d) CD103^+^CD11c^+^INOS^+^DCs (iNOS^+^DCs) analysis: D1: Nor group; D2: Nor + SSP group; D3: DSS group; D4: DSS + SSP group; D5: statistical analysis of CD103^+^CD11c^+^INOS^+^DCs proportion. (e) CD103^+^CD11c^+^MHC-II^+^DCs (MHC-II^+^DCs) analysis: E1: Nor group; E2: Nor + SSP group; E3: DSS group; E4: DSS + SSP group; E5: statistical analysis of CD103^+^CD11c^+^INOS^+^DCs proportion. (f) CD11c^+^CD103^+^CD324^+^DCs (E-cadherin^+^DCs) analysis: F1: Nor group; F2: Nor + SSP group; F3: DSS group; F4: DSS + SSP group; F5: statistical analysis of CD11c^+^CD103^+^CD324^+^DCs proportion. (g) CD103^+^CD11c^+^CD107b^+^DCs (Mac-3^+^ DCs) analysis: G1: Nor group; G2: Nor + SSP group; G3: DSS group; G4: DSS + SSP group; G5: statistical analysis of CD103^+^CD11c^+^CD107b^+^DCs proportion. (h) CD103^+^ CD11c^+^CD115^+^DCs (GM-CSFR^+^DCs) analysis: H1: Nor group; H2: Nor + SSP group; H3: DSS group; H4: DSS + SSP group; H5: Statistical analysis of group; H5: statistical analysis of CD103^+^CD11c^+^CD115^+^DCs proportion; data are expressed as mean ± SEM (*n* = 8). ^*∗*^*p* *<* *0*.05 and ^*∗∗*^*p* < 0.01 versus Nor group; ^#^*p* < 0.05 and ^##^*p* < 0.01 versus DSS group.

Inflammatory DCs (infDCs) are a unique subpopulation of DCs during inflammation mainly derived from monocytes at inflammatory parts [27]. In this study, the percentages of CD103^+^CD11c^+^iNOS^+^ (iNOS^+^DCs) (Figure 3D1-D5), CD103^+^CD11c^+^TNF-*α*^+^ (TNF-*α*^+^ DCs) (Figure 3C1-C5), CD11c^+^CD103^+^CD324^+^ (E-cadherin^+^ DCs) (Figure 3F1-F5), CD103^+^CD11c^+^MHC-II^+^ (MHC-II^+^DCs) (Figure 3E1-E5), CD103^+^CD11c^+^CD107b^+^ (Mac-3^+^DCs) (Figure 3G1-G5), and CD103^+^CD11c^+^CD115^+^ (GM-CSFR^+^DCs) (Figure 3H1-H5) were significantly higher in the DSS group than those of the Nor and Nor + SSP groups. After 7 consecutive days of SSP treatment, iNOS^+^DCs (Figure 3D1-D5), TNF-*α*^+^DCs (Figure 3C1-C5), E-cadherin^+^ DCs (Figure 3F1-F5), MHC-II^+^DCs (Figure 3E1-E5), and GM-CSFR^+^DCs (Figure 3H1-H5) were significantly decreased in the DSS + SSP group compared with the DSS group. These results suggest that SSP significantly inhibits the differentiation of infDCs in colitis mice with SKYD syndromes.

### 3.4. SSP Regulates the Composition of Gut Microbiome in Colitis Mice with SKYD Syndromes

**Figure 4:**
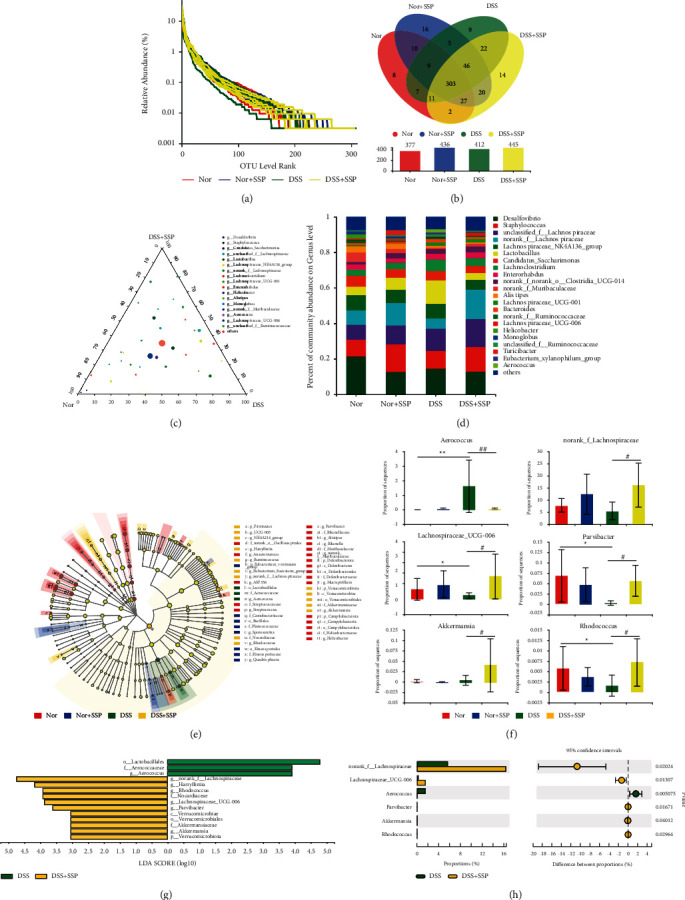
SSP regulates the composition of the gut microbiome in colitis mice. (a) Rank-abundance curves at the operational taxonomic unit (OTU) level. (b) The Venn diagram depicts OTUs that differed in different groups. (c) Ternary diagram analysis at the genus level in the Nor group, DSS group, and DSS + SSP group. (d) Community bar plot analysis at the genus level in different groups. (e) Taxonomic cladogram obtained from LEfSe analysis at phylum to genus level. (f) Differential analysis of *Aerococcus, norank_f_Lachnospiraceae, Lachnospiraceae_UCG-006, Parvibacter, Akkermansia,* and *Rhodococcus* among different groups. (g) Linear discriminant analysis (LDA) score between the DSS and DSS + SSP groups at phylum to genus level. (h) Differential analysis compared to the DSS and DSS + SSP groups at the genus level. Data were presented as means ± SEM (*n* = 3). ^*∗*^*p* < 0.05 and ^*∗∗*^*p* < 0.01 versus Nor group; ^#^*p* < 0.05 and ^##^*p* < 0.01 versus DSS group.

After sequence pumping leveling and OTU clustering, OTUs with 97% similarity were selected for bioinformatic statistical analysis. The relative abundance curves (Figure 4(a)) of all samples at the OTU level were obtained, and the smoother descending curve in the figure indicates the higher species diversity of the samples. The number of OTUs common to the samples between groups was counted, and 298 OTUs overlapped in each group (Figure 4(b)). The ternary-phase plot (Figure 4(c)) and community bar plot (Figure 4(d)) were used to analyze the effects of SSP on the composition of the GM and the dominant species at the genus levels in colitis with SKYD syndromes. The community bar plot analysis at the genus levels showed that the relative abundance of *norank_f_Lachnospiraceae*, *norank_f_Muribaculaceae*, *Lachnospiraceae_UCG-006*, and *Monoglobus* decreased in the DSS group, while *Lactobacillus*, *Lachnoclostridium*, *Bacteroides*, *Aerococcus,* and *unclassified_f_Ruminococcaceae* increased, compared with the Nor and DSS + SSP groups. Meanwhile, *Aerococcus*, *Lactobacillus*, *Lachnoclostridium*, and *Lachnospiraceae_NK4A136_group* were the dominant species in the DSS group, and *norank_f_Lachnospiraceae*, *Lachnospiraceae_UCG-006*, *unclassified_f_Lachnospiraceae*, *Enterorhabdus*, and *Staphylococcus* were the dominant species in the DSS + SSP groups. These data suggest SSP can improve the GM composition in colitis mice with SKYD syndromes.

The imbalance of GM homeostasis leads to the colonization and invasion of opportunistic pathogens in the gut, which increases the risk of host immune response and promotes the development of IBD [28]. In order to further investigate the effect of SSP on specific GM in colitis mice, the nonparametric factorial Kruskal–Wallis sum-rank test and Wilcoxon rank-sum test of Lefse software were used to analyze communities with significant differences in species abundance on the genus level (Figure 4(f)) among the Nor, Nor + SSP, DSS, and DSS + SSP groups. Phylum-to-genus-based evolutionary branching plots (Figure 4(e)) showed the structure of microbiota and their dominant species in each group. The histograms of dominant communities (Figure 4(g)) in the DSS and DSS + SSP groups (LDA score >2.0, *p* < 0.05) showed that *o_Lactobacillales*, *f_ Aerococcaceae*, and *g_Aerococcus* were the dominant species in the DSS group, and *g_norank_f_Lachnospiraceae*, *g_Harryflintia*, *g_Rhodococcus*, *f_Nocardiaceae*, *g_Lachnospiraceae_UCG-006*, *g_Parvibacter*, *c_Verrucomicrobiae*, *o_Verrucomicrobiales*, *f_Akkermansiaceae*, *g_Akkermansia*, and *p_Verrucomicrobiota* were the dominant species in the DSS + SSP groups. In addition, the rank-sum test at the genus level showed (Figure 4(h)) that compared to the DSS group, the relative abundance of *norank_f_Lachnospiraceae*, *Lachnospiraceae_UCG-006*, *Parvibacter*, *Akkermansia*, and *Rhodococcus* in the DSS + SSP groups increased significantly, and *Aerococcus* significantly decreased. The above data analysis indicated that SSP could regulate some specific GM in colitis mice with SKYD syndromes.

### 3.5. SSP Improves Fecal Metabolome in Colitis Mice with SKYD Syndromes

**Figure 5:**
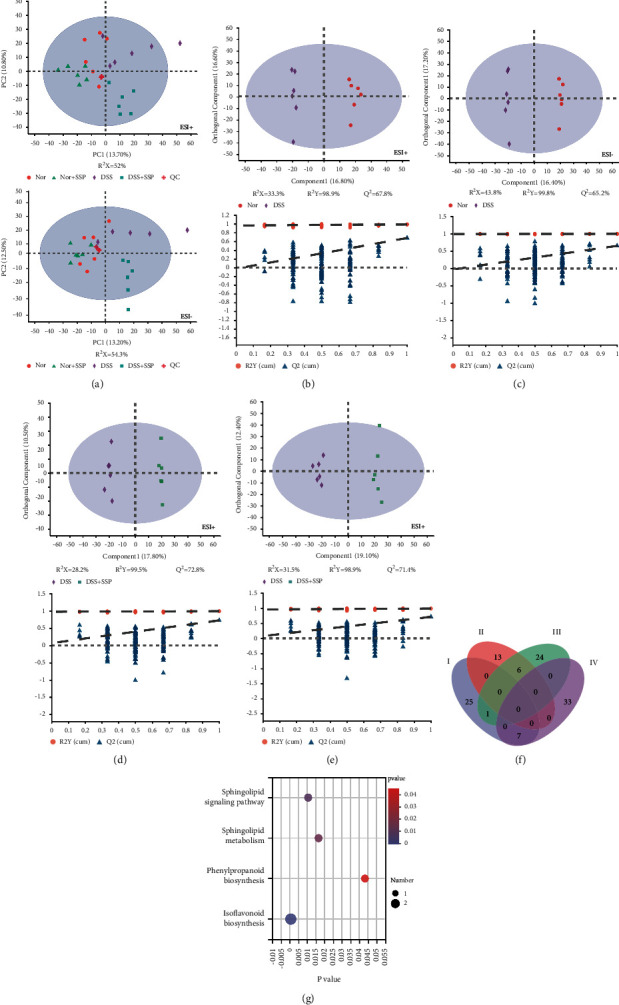
SSP treatment-induced changes in fecal metabolome. (a) Score plots of PCA between the Nor, Nor + SSP, DSS, DSS + SSP, and QC groups. (b, c) Score plots of OPLS-DA between the Nor and DSS groups and the corresponding coefficient of loading plots. (d, e) Scores plots of OPLS-DA between the DSS and DSS + SSP groups and the corresponding coefficient of loading plots. (f) Numbers of differential metabolites between the Nor, DSS, and DSS + SSP groups (Venn diagram). I: Elevated levels in the DSS group vs. Nor group; II: decreased levels in the DSS group vs. Nor group; III: elevated levels in the DSS + SSP group vs. DSS group; IV: decreased levels in the DSS + SSP group vs. DSS group. (g) KEGG enrichment analysis showed the pathway name of 13 common differential metabolites between the Nor, Nor + SSP, DSS, DSS + SSP groups (*p* < 0.05).

The regulation mechanisms of GM-associated metabolites affect the intestinal immune system and their interactions in pathological conditions such as IBD [29]. Differential metabolite addition, deletion, and metabolic pathway regulation can be used to treat IBD. This study used the LC-MS platform to extract raw mouse fecal mass spectrometry data, including QC and detection samples. According to the PCA model, there was a clear overlap between the Nor and Nor + SSP group, while the separation between the Nor, DSS, and DSS + SSP groups was unclear (Figure 5(a)). Therefore, we performed OPLS-DA to further visualize the metabolic alterations occurring between the Nor group and DSS group as well as between the DSS group and the DSS + SSP group. The OPLS-DA models showed significant distinctions in metabolomic data between the Nor group and the DSS group (Figures 5(b) and 5(c)) as well as between the DSS group and the DSS + SSP group (Figures 5(d) and 5(e)). These results indicate that the OPLS-DA models were robust. Metabolites with a *p* < 0.05 and VIP >1 between the Nor and DSS groups or between the DSS + SSP and DSS groups were considered to be differential metabolites. Importantly, the Venn diagrams (Figure 5(f)) identified 13 common differential metabolites that significantly changed in the DSS group as compared to the Nor group and significantly changed in the opposite direction in the DSS + SSP group as compared to the DSS group (Table 1). Finally, the plot of the Kyoto Encyclopedia of Genes and Genomes (KEGG) enrichment analysis (Figure 5(g)) of 13 common differential metabolites revealed that sphingolipid signaling pathway, sphingolipid metabolism, phenylpropanoid biosynthesis, and isoflavonoid biosynthesis were significantly enriched KEGG pathways.

### 3.6. Pearson and Spearman's Correlation

**Figure 6:**
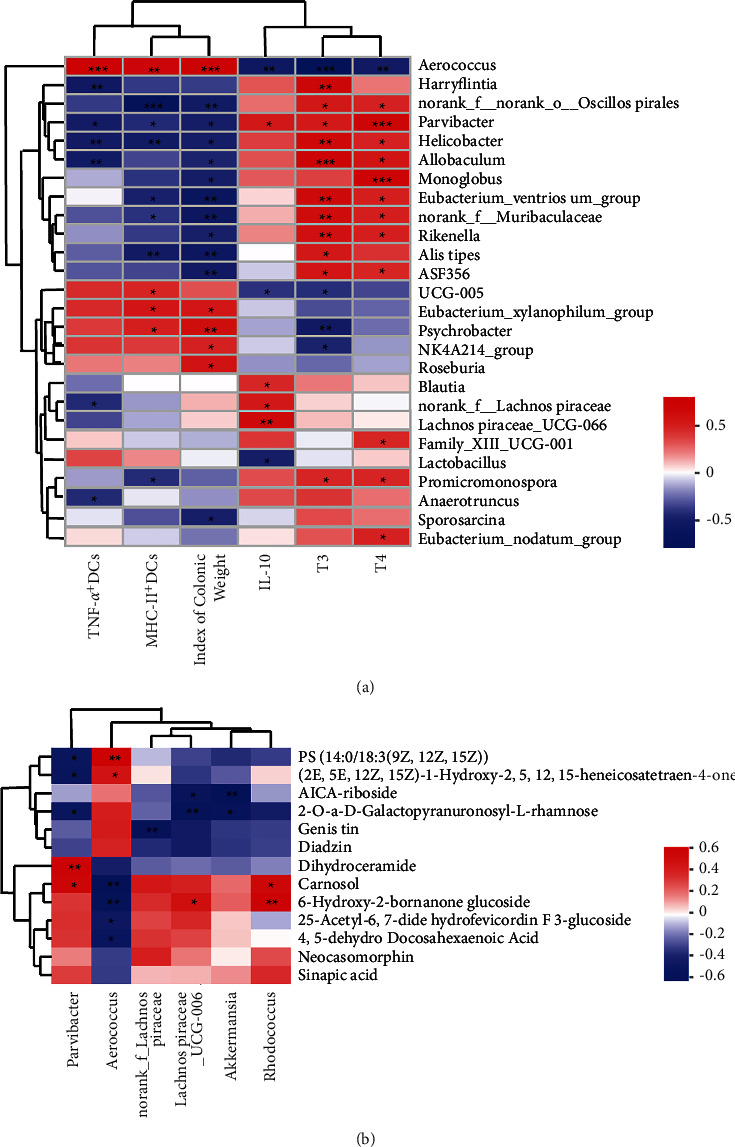
Pearson and Spearman's correlation. (a) Spearman's correlation heatmap of FT3, FT4, IL-10, index of colonic weight, TNF-*α*^+^DCs, MHC-II^+^DCs, and gut microbiota. (b) Pearson's correlation heatmap of *Parvibacter, Aerococcus, norank_f_Lachnospiraceae, Lachnospiraceae_UCG-006, Akkermansia, Rhodococcus,* and 13 common differential metabolites.

When the host is in the process of immune activation, inflammation, and related changes in the GM, the influence of the host's innate immunity on the GM also increases, and the presence of specific bacteria may also affect the disease phenotype, such as IBD [30]. The present results suggested that SSP alleviates colitis mice with SKYD syndromes in association with GM, fecal metabolites, infDCs, and inflammatory cytokines. The Spearman correlation heat map (Figure 6(a)) further found the correlation between the environmental factors and the relative abundance of the top 30 species at the genus levels. Moreover, the Pearson correlation heat map (Figure 6(b)) showed the correlation between 6 specific bacteria and 13 common differential metabolites. Of note, SSP alleviated colitis mice with SKYD syndromes closely correlated with inflammatory cytokines, infDCs, GM, and fecal metabolites; *Aerococcus* were positively correlated with TNF-*α*^+^ DCs, MHC-II^+^ DCs, index of colonic weight, PS (14 : 0/18 : 3(9Z,12Z,15Z)), (2E,5 E,12Z,15Z)-1-hydroxy-2,5,12,15-heneicosatetraen-4-one, and negatively correlated with IL-10, FT3, FT4, carnosol, 6-hydroxy-2-bornanone glucoside, 25-acetyl-6,7-didehydrofevicordin F 3-glucoside, 4,5-dehydro docosahexaenoic acid; *Lachnospiraceae_UCG-006* was positively correlated with IL-10 and 6-hydroxy-2-bornanone glucoside, and negatively correlated with AICA-riboside and 2-O-a-D-galactopyrannosyl-L-rhamnose; *norank_f_Lachnospiraceae* was positively correlated with IL-10 and negatively correlated with TNF-*α*^+^ DCs and genistin; *Parvibacter* were positively correlated with IL-10, FT3, FT4, dihydroceramide and carnosol, and negatively correlated with TNF-*α*^+^ DCs, MHC-II^+^ DCs, index of colonic weight, PS (14 : 0/18 : 3(9Z,12Z,15Z)), (2E,5 E,12Z,15Z)-1-Hydroxy-2,5,12,15-heneicosatetraen-4-one, and 2-O-a-D-galactopyranuronosyl-L-rhamnose.

### 3.7. SSP Suppresses TLR4/NF-*κ*B Signaling Pathway in Colitis Mice with SKYD Syndromes

**Figure 7:**
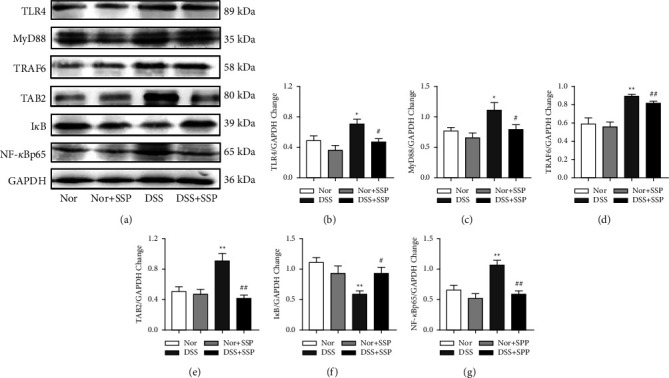
SSP suppresses TLR4/NF-*κ*B signaling pathway in colitis mice. (a) Expression of TLR4/NF-*κ*B signaling pathway proteins (TLR4, MyD88, TRAF6, TAB2, I*κ*B, and NF-*κ*Bp65) by Western blot. (b-g) Quantitative analysis of TLR4 (b), MyD8 (c), TRAF6 (d), TAB2 (e), I*κ*B (f), and NF-*κ*Bp65 (g). Data are expressed as mean ± SEM (*n* = 8). ^*∗*^*p* *<* 0.05 and ^*∗∗*^*p* < 0.01 versus Nor group; ^#^*p* < 0.05 and ^##^*p* < 0.01 versus DSS group.

Under steady-state conditions, classical pattern recognition receptors promote immune development, enhance immunity, and maintain beneficial host-microbe symbiosis by recognizing microbial-associated molecular patterns [31]. In order to observe this process in colitis mice, Western blotting was used to detect the levels of the TLR4/NF-*κ*B signaling molecules. After 7 consecutive days of SSP treatment, the levels of proteins in the TLR4/NF-*κ*B signaling pathway in the colonic mucosa were significantly higher in the DSS group than those in the Nor, Nor + SSP, and DSS + SSP groups, including TLR4 (Figures 7(a) and 7(b)), MyD88 (Figures 7(a) and 7(c)), TRAF6 (Figures 7(a) and 7(d)), TAB2 (Figures 7(a) and 7(e)), and NF-*κ*Bp65 (Figures 7(a) and 7(g)), while the level of I*κ*B (Figures 7(a) and 7(f)) proteins was significantly lower. These data further suggest that SSP can inhibit the TLR4/NF-*κ*B signaling pathway in colitis mice with SKYD syndromes.

## 4. Discussion

In the present study, the rhubarb + hydrocortisone + DSS-induced colitis mice showed symptoms such as emaciation, dispiritedness, hair erect and back arched, huddle, loose stools, and the rate of body weight change, and the levels of FT3, FT4, and TESTO decreased significantly, which is in line with the clinical symptoms of SKYD. These colitis mice also showed a shorter colonic length, increased colonic weight and index of colonic weight, DAI, and pathological damage score, as well as increased levels of pro-inflammatory cytokines TNF-*α*, IL-1*β*, IL-6, and IL-12p70, and decreased the level of anti-inflammatory cytokine IL-10. Colonic pathological damage, mucosal inflammatory response, and imbalance of pro-inflammatory/anti-inflammatory cytokines were considered to be the characteristics of intestinal inflammatory changes in experimental colitis [24]. The above data suggest that the animal model of colitis with SKYD syndromes was successfully replicated. Importantly, 7 days of consecutive treatment with SSP reversed this process to some extent, suggesting that SSP effectively ameliorates UC with SKYD syndrome in mice.

Next, we found that colitis mice with SKYD syndromes exhibited immune hyperactivity (iNOS^+^ DCs, TNF-*α*^+^ DCs, E-cadherin^+^ DCs, MHC-II^+^ DCs, Mac-3^+^ DCs, and GM-CSFR^+^ DCs were abnormally activated). DCs that differentiate from monocytes in inflammatory tissues and are stored in peripheral tissues and lymphoid organs are called infDCs [32]. Initially, infDCs were identified as MHC-II, CD11b, CD11c, F4/80, and Ly6C DCs [33]. Yet, recent studies have found that infDCs highly express TNF-*α*, iNOS, E-cadherin, CD206, CD115/GM-CSFR, CD107b/Mac-3, FCERI, and CD64 [27, 34–36]. InfDCs can induce Th1, Th2, Th17, and CD8^+^T-cell responses and promote the release of TNF-*α*, IL-1*β*, IL-6, L-12, and IL-23 [27, 32–34]. In patients with UC, CD103^+^ DCs have a crucial role in the pathogenesis, preferentially by inducing Th1/Th2/Th17 responses rather than generating Treg cells [37]. As typical indicators of infDCs, the inhibition of TNF-*α*^+^ DCs, E-cadherin^+^ DCs, and pro-inflammatory cytokines in colitis mice has been considered a potential therapeutic target [36]. In this study, we found that SSP can reduce the levels of iNOS^+^ DCs, TNF-*α*^+^ DCs, E-cadherin^+^ DCs, MHC-II^+^ DCs, and GM-CSFR^+^ DCs in colitis mice with SKYD syndromes. The above results suggest that the therapeutic effect of SSP on intestinal inflammation was potentially realized by inhibiting the differentiation of infDCs.

Owing to a complex network of interactions between host immunity, microbes, and microbial metabolites govern intestinal homeostasis, making the pathogenesis and therapeutic mechanism of IBD more challenging. The discovery of microbiome, transcriptome, and systems biology approaches has provided a reliable way to elucidate complex mechanisms [38, 39]. In recent years, the dialogue between GM and host immunity has become a hot spot for IBD treatment [40]. It is known that Chinese herbal medicine has a therapeutic role through GM and immunity; yet, the exact mechanism is still unclear. On the one hand, Chinese herbal ingredients affect the metabolic activities of amino acids and lipids by changing the abundance and diversity of the GM to regulate host immunity. On the other hand, Chinese herbal ingredients are transformed and metabolized by intestinal flora to produce new functionally active products and improve the bioavailability and efficacy of drugs [41].

The causal relationship between specific microbes and IBD has not been fully confirmed. Some studies found that the reduced relative abundance of beneficial microbes and the enrichment of pathogenic microbes in the gut is associated with the occurrence and development of IBD [42, 43]. In the present study, *Aerococcus*, *Lactobacillus*, *Lachnoclostridium*, and *Lachnospiraceae_NK4A136_group* were the dominant bacteria of colitis mice with SKYD syndromes. Some studies have shown that *Aerococcus* can effectively aggravate DSS-induced colitis [44]. Moreover, studies found that *Lachnoclostridium* are enriched in the gut of UC with SKYD syndromes [45], while *Lactobacillus* are enriched in the stool of IBD compared to healthy people [46]. This evidence indicates that the pathogenesis of colitis mice with SKYD syndromes is related to the enrichment of pathogenic microbes. Herein, we reported for the first time that SSP inhibits the enrichment of *Aerococcus* and increases the relative abundance of *norank_f_Lachnospiraceae*, *Lachnospiraceae_UCG-006*, *Parvibacter*, *Akkermansia*, and *Rhodococcus*. Meanwhile, the correlation analysis showed that *Aerococcus* were positively correlated with TNF-*α*^+^ DCs, MHC-II^+^ DCs, and colonic weight index and negatively correlated with IL-10, FT3, and FT4; *norank_f_Lachnospiraceae*, *Rhodococcus,* and *Lachnospiraceae_UCG-006* were positively correlated with IL-10; *Parvibacter* were positively correlated with IL-10, FT3, and FT4, but negatively correlated with TNF-*α*^+^ DCs, MHC-II^+^ DCs, and index of colonic weight. Some studies indicated that the increased abundance of *Lachnospiraceae_UCG-006* improves the differentiation of Treg cells and restores the secretion levels of IL-10 in experimental colitis [47]. The increased abundance of *norank_f_Lachnospiraceae* is also confirmed to exert an anti-inflammatory role by regulating the interaction between infDCs and Tregs [48]. As pathogenic microbes, *Aerococcus* effectively aggravates experimental colitis by improving Th2 cell response, decreasing unsaturated fatty acid levels, and increasing arachidonic acid metabolism [44]. Our data indicated that SSP inhibits the differentiation of TNF-*α*^+^ DCs and MHC-II^+^ DCs, and increases the secretion levels of IL-10, further rebuilding the balance of GM in colitis mice with SKYD syndromes. Yet, the relationship between SSP, infDCs, and GM needs to be further evaluated in future studies.

Increasing evidence showed that lipid metabolism mediated by GM is involved in the development, maturation, and function of DCs, including bile acids, short-chain fatty acids, specialized pro-resolving mediators, lysophospholipids, endogenous cannabinoids, oxysterols, and sphingolipids [12, 49, 50]. In the present study, colitis mice with SKYD syndromes showed significant dysregulation of metabolites, while 13 metabolites were effectively reversed after SSP treatment. KEGG enrichment analysis of 13 common differential metabolites further demonstrates that the sphingolipid signaling pathway, sphingolipid metabolism, and phenylpropanoid biosynthesis were significantly enriched. Some studies have confirmed that sinapic acid significantly ameliorates acetic acid-induced UC in rats by suppressing cytokines TNF-*α* and IL-6 in colonic tissues [51]. Sphingolipids have an indispensable role in intestinal inflammation [52–54], including ceramide, ceramide 1-phosphate, sphingomyelin, and sphingosine 1-phosphate. Innate immunity cell receptors can be activated by the above sphingolipid to affect the physiological and pathological processes of IBD, such as TLR, PPAR*α*/*γ*, AhR, *G*protein-coupled receptors, and endogenous cannabinoids [52, 55]. Likewise, in our study, Spearman's analysis showed that dihydroceramide was positively correlated with *Parvibacter*. The growth of *Parvibacter* in mice fecal samples improves hepatic lipid metabolism and meta-inflammation in HFD-induced obese mice [56]. Therefore, we hypothesized that SSP could regulate the disorders in the sphingolipid signaling pathway and sphingolipid metabolism by improving dysbiosis in GM.

Nevertheless, the crosstalk mechanism among GM, microbiota metabolites, and infDCs remains unclear. In the present study, after the SSP treatment of colitis mice with SKYD syndromes for 7 days, TLR4, MyD88, TRAF6, TAB2, and NF-*κ*Bp65 proteins were inhibited, and I*κ*B was activated in colonic mucosal tissues, which suggested that the therapeutic effect of SSP on experimental colitis is closely related to the TLR4/NF-*κ*B signaling pathway. The pro-inflammatory role of the intestinal TLR4/NF-*κ*B signaling cascade has been demonstrated in IBD [57]. Excessive activation of TLR4 in intestinal epithelial cells induces GM disturbance and increased susceptibility to colitis [58]. Indeed, multiple pattern recognition receptors (PRRs) expressed by antigen-presenting cells are activated inflammatory signals by identifying microbe-associated molecular patterns or metabolites, such as lipopolysaccharides, short-chain fatty acids, and fatty acids, thereby achieving immune alert or pathogen clearance [59, 60]. Due to abnormal DC activation evident in the IBD, these infDCs significantly express high levels of maturity marker CD40 and pro-inflammatory cytokines IL-12 and IL-6 associated with DCs in colonic mucosal tissues, thereby inducing strong adaptive immunity [61]. It is worth emphasizing that the regulated state of infDCs, GM, and fecal metabolites and inhibited activation of the PI3K/Akt signaling pathway were synchronously found when SSP alleviated the pathological colonic injury in colitis mice with SKYD syndromes.

## 5. Conclusions

**Figure 8:**
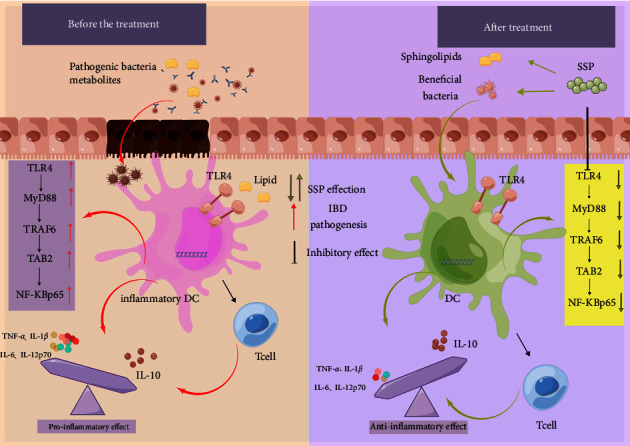
Schematic mechanism of SSP-treated mice colitis with SKYD syndrome via gut microbiota, fecal metabolites, inflammatory dendritic cells, and TLR4/NF-*κ*B pathway.

In summary, GM, fecal metabolites, infDCs, and TLR4/NF-*κ*B signaling have important roles in the effects of SSP against colitis mice with SKYD syndromes. The therapeutic effect of SSP on colitis with SKYD syndromes might be related to the inhibition of the TLR4/NF-*κ*B signaling pathway, which corrects the immune interactions of GM or microflora metabolites with infDCs, and then maintains the symbiotic relationship between the microflora and the host (Figure 8). Yet, the interactions of the microbiota-metabolism-immune axis of SSP need to be further explored in future studies. GM depletion and fecal transplantation could be used to validate SSP's ability to modulate the fecal sphingolipid metabolism by improving the dysfunction of the GM community. Of course, we clearly recognized that we should isolate infDCs from the tissue, use fecal supernatant after SSP treatment to treat their cells, and finally extract the infDC suspension to analyze the activation of TLR4/NF-*κ*B signal, which we plan to do in our future study.

## Figures and Tables

**Table 1:** The common differential metabolites in the fecal sample between the Nor, DSS, and DSS + SSP groups.

NO	Rt (min)	m/z	Formula	Metabolites	VIP		FC		Trend		Pathway
					D vs N	S vs D	D vs N	S vs D	D vs N	S vs D	
1	4.01	223.06	C_11_H_12_O_5_	Sinapic acid	2.11	2.31	0.66	1.60	↓^#^	↑^*∗*^	c
2	9.38	325.22	C_22_H_30_O_2_	4,5-Dehydro docosahexaenoic acid	2.22	2.02	0.65	1.48	↓^##^	↑^*∗∗*^	-
3	9.31	750.43	C_38_H_68_NO_10_P	PS(14 : 0/18 : 3(9Z,12Z,15Z))	3.06	2.29	1.54	0.74	↑^##^	↓^*∗∗*^	-
4	7.75	363.25	C_21_H_34_O_2_	(2E,5 E,12Z,15Z)-1-hydroxy-2,5,12,15-heneicosatetraen-4-one	2.00	1.50	1.35	0.81	↑^##^	↓^*∗*^	-
5	5.93	751.34	C_35_H_52_N_6_O_10_	Neocasomorphin	1.91	2.15	0.74	1.49	↓^#^	↑^*∗∗*^	-
6	1.12	321.08	C_12_H_20_O_11_	2-O-a-D-Galactopyranuronosyl-L-rhamnose	1.79	1.66	1.21	0.81	↑^#^	↓^*∗*^	-
7	0.78	239.08	C_9_H_14_N_4_O_5_	AICA-riboside	1.94	2.29	1.28	0.62	↑^##^	↓^*∗*^	-
8	3.73	433.11	C_21_H_20_O_10_	Genistin	2.00	1.76	1.26	0.79	↑^##^	↓^*∗*^	d
9	5.21	369.13	C_16_H_26_O_7_	6-Hydroxy-2-bornanone glucoside	1.87	2.57	0.75	1.53	↓^#^	↑^*∗∗*^	-
10	6.26	331.19	C_20_H_26_O_4_	Carnosol	1.96	2.36	0.79	1.34	↓^##^	↑^*∗∗*^	-
11	5.81	705.35	C_37_H_52_O_13_	25-Acetyl-6,7-didehydrofevicordin F 3-glucoside	2.40	2.15	0.72	1.37	↓^##^	↑^*∗*^	-
12	5.65	330.30	C_19_H_39_NO_3_	Dihydroceramide	2.87	1.61	0.50	1.43	↓^##^	↑^*∗*^	a, b
13	3.34	417.12	C_21_H_20_O_9_	Daidzin	1.92	2.19	1.40	0.66	↑^#^	↓^*∗∗*^	d

Nor, DSS, and DSS + SSP (*n* = 6 per group) groups. ^#^*p* < 0.05 as compared to the Nor group; ^##^*p* < 0.01 as compared to the Nor group; ^*∗*^*p* < 0.05 as compared to the DSS group; ^*∗∗*^*p* < 0.01 as compared to the DSS group; ↑, content increased; ↓, content decreased; vs., versus; N, Nor group; D, DSS group; S, SSP group. (a) Sphingolipid metabolism. (b) Sphingolipid signaling pathway. (c) Phenylpropanoid biosynthesis. (d) Isoflavonoid biosynthesis.

## Data Availability

The data presented in this study can be obtained from the corresponding author upon request.

## Abbreviations

SSP: Sishen pill
IBD: Inflammatory bowel disease
UC: Ulcerative colitis
SKYD: Spleen-kidney-yang deficiency
DSS: Dexglycan sodium sulfate
DAI: Disease activity index
FT3: Triiodothyronine
FT4: Free tetraiodothyronine
TESTO: Testosterone
TNF: Tumor necrosis factor
IL: Interleukin
DCs: Dendritic cells
InfDCs: Inflammatory dendritic cells
GM: Gut microbiota
OTU: Operational taxonomic units
QC: Quality control
PCA: Principal component analysis
OPLS-DA: Orthogonal partial least squares discriminant analysis
VIP: Variable importance in the projection
LDA: Linear discriminant analysis
KEGG: Kyoto Encyclopedia of Genes and Genomes.
